# Hippocampal representations for deep learning on Alzheimer’s disease

**DOI:** 10.1038/s41598-022-12533-6

**Published:** 2022-05-21

**Authors:** Ignacio Sarasua, Sebastian Pölsterl, Christian Wachinger

**Affiliations:** 1 Artificial Intelligence in Medical Imaging (AI-Med), Department of Child and Adolescent Psychiatry, Ludwig-Maximilians-Universität, Waltherstr. 23, 80337 Munich, Germany; 2 Klinikum Rechts der Isar, Technical University of Munich, Ismaninger Str. 22, 81675 Munich, Germany

**Keywords:** Neuroscience, Cognitive ageing, Cognitive neuroscience, Computer science, Brain

## Abstract

Deep learning offers a powerful approach for analyzing hippocampal changes in Alzheimer’s disease (AD) without relying on handcrafted features. Nevertheless, an input format needs to be selected to pass the image information to the neural network, which has wide ramifications for the analysis, but has not been evaluated yet. We compare five hippocampal representations (and their respective tailored network architectures) that span from raw images to geometric representations like meshes and point clouds. We performed a thorough evaluation for the prediction of AD diagnosis and time-to-dementia prediction with experiments on an independent test dataset. In addition, we evaluated the ease of interpretability for each representation–network pair. Our results show that choosing an appropriate representation of the hippocampus for predicting Alzheimer’s disease with deep learning is crucial, since it impacts performance and ease of interpretation.

## Introduction

Alzheimer’s disease (AD) is a progressive brain disorder characterized by a gradual and irreversible degradation of cognitive functions over time. Given the correlation between neuron loss and brain atrophy measured in magnetic resonance imaging (MRI)^1^, in vivo neuroimaging has become invaluable for studying trajectories of pathophysiological change in AD. Machine learning (ML) methods have been proposed to predict diagnosis and prognosis based on these AD related changes observed in MRI sequences^2,3^. In particular, a focus on the hippocampus has been set due to its important role for the creation of new memory and measurable atrophy in MRI. In order to capture finer changes of this subcortical structure, shape vectors have been used given their higher sensitivity to anatomical variations compared to volume measures, e.g. early morphological changes in subnuclei^4–10^.

More recently, deep learning methods have been proposed for predicting AD diagnosis^11^ and prognosis^12–15^ from MRI. Instead of working with handcrafted features, deep learning offers the ability to learn representations that are optimal for the given task^16^. Nevertheless, a format needs to be selected to serve as input to the neural network. A *region of interest* (ROI) of the scan around the hippocampus is one possibility^17–20^, but does not provide any explicit geometric information or localization, which is available from segmentations of the hippocampus. Recent advances in the field of geometric deep learning^21^ have provided novel neural network architectures that work on geometric objects. For AD prediction based on the shape of the hippocampus, inputs to neural networks have been binary volumetric masks^22,23^, triangular meshes^24–26^, and point clouds^27–29^. *Volumetric masks* are defined on a pixel grid, hence typical CNN architectures can be used and it is easy to extend them to *volumetric texture* representations of the hippocampus, by adding the MRI intensity values. *Point clouds* are a simple shape representation that consist of a set of 3D points that are typically sampled from the surface of the hippocampus. *Meshes* have connectivity information, which establishes a more comprehensive representation of the underlying anatomical surface that supports different levels of granularity.

Choosing the input representation to the neural net is important as it also determines the type of network architectures that are applicable. Yet, no comprehensive comparison is available to date. A direct comparison of published results for each representation would not meaningful, as studies vary in the number of subjects, dataset splits, and evaluation procedure. Importantly^11^, have recently surveyed 32 deep learning methods for AD prediction and found that half of the studies were subject to one or more sources of data leakage. Hence a rigorous comparison the different hippocampus representations is necessary. In addition limiting the analysis to a single structure (instead of the whole brain) provides a fairer comparison between representations since the extension to multi-structural data is not as straight forward for all of them (e.g. meshes). Furthermore, the comparison should not only be limited to a performance evaluation, but should also assess the interpretability of different representations, since this is a key factor for clinical acceptance. Finally, different neuroimaging tools (such as FSL and FreeSurfer) exist for the segmentation of the hippocampus that vary in the type of output (masks and meshes) and their impact on deep learning has not been evaluated yet.

In this study, we perform a rigorous comparison of five hippocampus representations that span a continuum from raw image intensities, over the usage of a hybrid of shape and texture, to pure shape representations with binary masks, meshes, and point clouds. For each representation, we select the corresponding state-of-the-art network architectures for the prediction of Alzheimer’s diagnosis and prognosis. For prognosis, we perform time-to-dementia prediction with survival analysis, which presents an appropriate statistical treatment of the progression to AD by explicitly modeling the timing of the event and by considering censoring and drop-out. We pay particular attention to the generation of the data splits to avoid confounding and data leakage. We further evaluate the generalizability of the models by evaluating on an independent dataset. While all our experiments are focused on hippocampus analysis, these finding can be extended to other anatomical structures and non-medical applications, such as object recognition.

Our results demonstrate the superior performance of meshes, compared to networks on point clouds and volumetric masks. They further demonstrate that hippocampus texture information contains helpful information to shape for diagnosis, but not for prognosis prediction. Finally, meshes show the best generalization to an independent cohort, and enabled the most meaningful identification of important Hippocampus regions for AD prediction. Finally, we demonstrate that predictions based on segmentations of the hippocampus created by FreeSurfer^30^ yield higher performance than those created by FSL FIRST^31^.

We believe this work can be of great interest since such an objective comparison between representations, i.e. all methods and networks evaluated by the same authority and on the same data, has not been done in the past.

**Figure 1:**
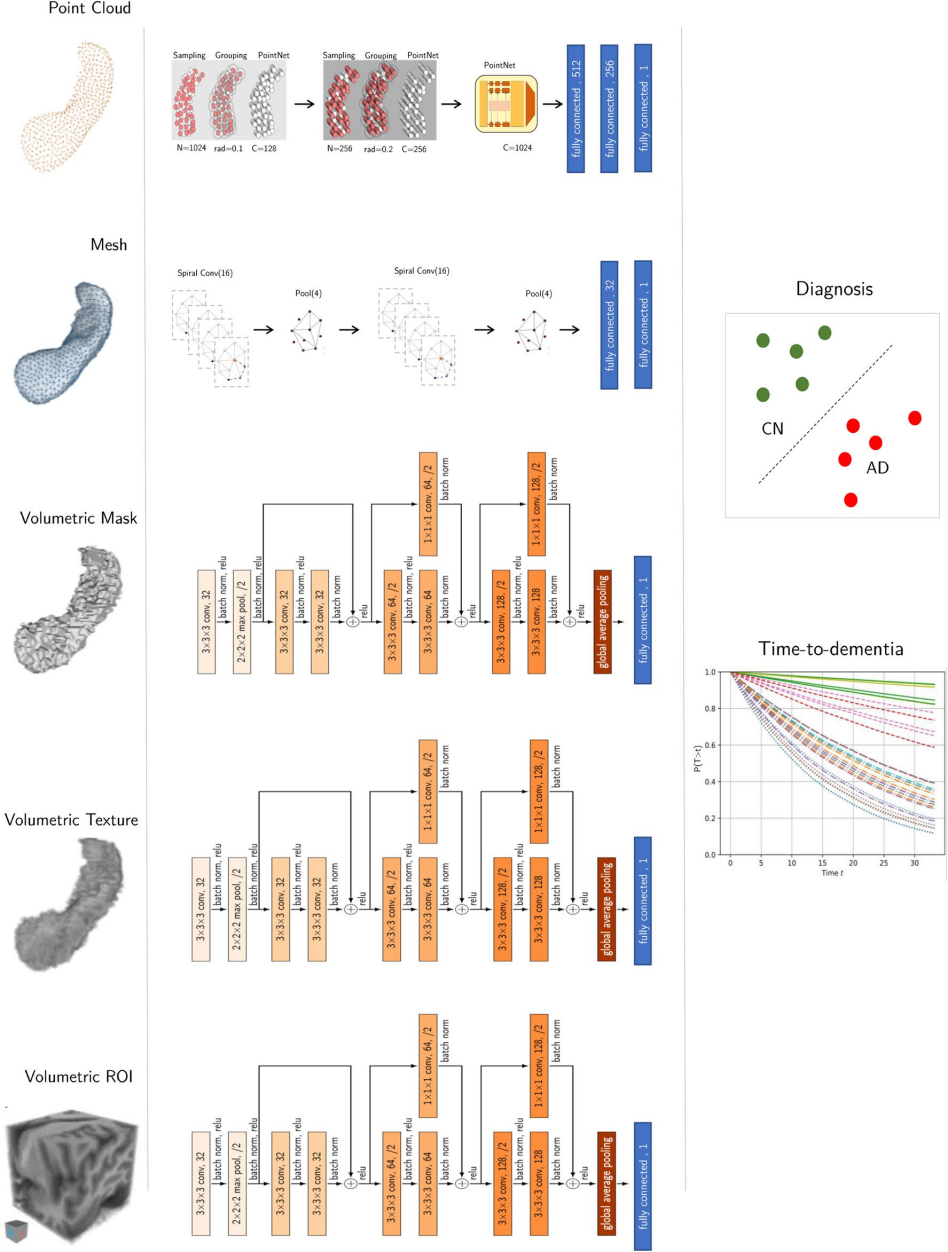
Evaluation scheme overview. Left column shows the different ways of representing the hippocampus. Middle column describes the different model architectures. The right column defines the two tasks that we evaluated in this study: diagnosis and time-to-dementia/prognosis.

## Hippocampus representations

Figure 1 shows an overview of this work. We consider five ways of representing the hippocampus: point clouds, meshes, volumetric masks, volumetric textures and volumetric ROIs. A *point cloud* is an un-ordered set of points, ${\mathbf {P}}= \{{\mathbf {p}}_1,\ldots,{\mathbf {p}}_N\}$, with ${\mathbf {p}}_i=[x_i,y_i,z_i]$ being the coordinates of point *i* on the surface of the hippocampus and *N* the number of points. A 3D *mesh*, ${\mathbf {M}} = ({\mathbf {V}},{\mathbf {E}}$) is defined by a set of vertices, ${\mathbf {V}}\in {\mathbb {R}}^{N\times 3}$, and edges, ${\mathbf {E}}$, that connect the vertices.

Volumetric representations encode the hippocampus on a fixed image grid. *Volumetric masks* encode the hippocampus as 3D binary mask—voxels belonging to the structure are set to one and zero otherwise. Similarly, *volumetric textures* are formed by keeping the grayscale values of the hippocampus voxels and setting the rest to zero. Finally, *volumetric ROIs* are 3D bounding boxes around the hippocampus, which do not only encode the hippocampus information but also its neighboring structures.

Point clouds, meshes, and volumetric masks only contain shape information, whereas volumetric ROIs only contain images intensities. Volumetric textures are a hybrid of both. We believe this selection of representations captures the whole spectrum of representing a 3D object

We train one deep neural network for each of the five different hippocampus representations to predict (i) dementia diagnosis (healthy/demented) and (ii) the time to dementia onset, respectively. As opposed to images, point clouds and meshes are not represented on a regular grid of discrete values and therefore common CNN operations such as *convolution* and *pooling* are not explicitly defined anymore. In particular, defining local neighborhood for a given vertex becomes challenging. After a comparison between state-of-the-art architectures for each representation (more details can be found in Table S5), we selected PointNet++^32^, SpiralNet++^33^ and ResNet^34^ for pointclouds, meshes and volumetric representations respectively, because they were the best performing models for their respective representation. In section “Networks” and Fig. 1, a description of each network can be found.

## Experiments

We evaluate the predictive performance for the classification experiments using balanced accuracy^35^ (BACC) and the area under the ROC Curve (AUC), and for the time-to-dementia experiments using the concordance index^36^ (c-index). The latter measures the rank-correlation between the predicted risk scores and the time until disease onset (where a c-index of 1.0 indicates a perfect model and 0.5 a completely random model).

**Figure 2:**
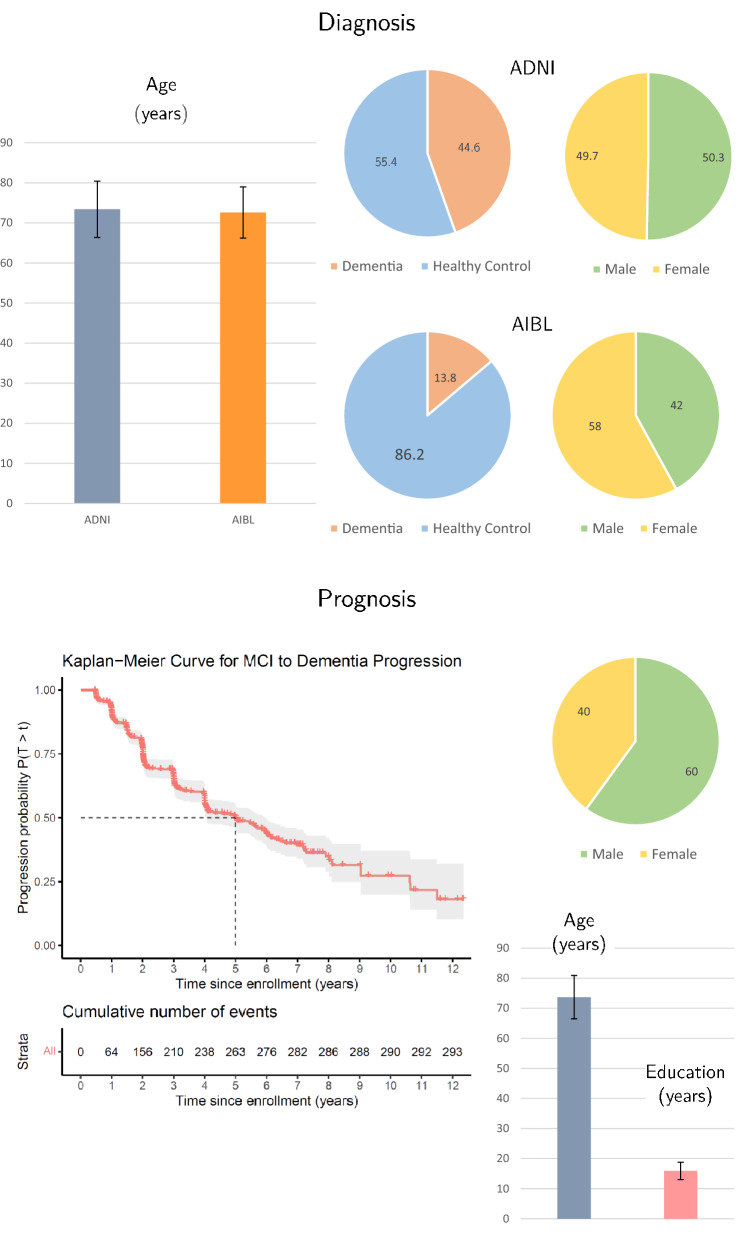
Top: Data statistics for the diagnosis task for ADNI and AIBL dataset. The data stratification strategy has been done according to three variables: age, sex and diagnosis. The total number of samples is 1505 for ADNI and 552 for AIBL. Bottom: Data statistics for the prognosis task. The data stratification across t the sets has been following 4 variables: time-to-dementia, sex, age and years of education

In our experiments, we use data from The Alzheimer’s Disease Neuroimaging Initiative (ADNI^37^) and The Australian Imaging, Biomarker & Lifestyle Flagship Study of Ageing (AIBL^38^), which are summarized in Fig. 2. We implemented the 5-fold cross-validation scheme described in section “Study population” on ADNI. The segmentations were obtained by using FSL-FIRST and FreeSurfer (see section “Data processing”). Moreover, we evaluated the performance of the five models trained on the ADNI data on an external patient population from AIBL. We also studied the effect of data augmentation as well as the interpretability of each method. Given that FreeSurfer does not provide registered meshes, we only provide mesh results for FSL. In addition, we note that the ROI extracted around the segmentation mask is the same for FSL and FreeSurfer and therefore is only reported once.

**Table 1:** Classification results (BACC: balanced accuracy, AUC: Area under the ROC curve) on ADNI and AIBL for different representations.

Representation	Network	ADNI BACC	ADNI AUC	AIBL BACC	AIBL AUC
**FSL**					
Point cloud	PointNet++	$0.755 \pm 0.014$	$0.821 \pm 0.017$	$0.727 \pm 0.005$	$0.802 \pm 0.007$
Mesh	SpiralNet++	$0.824 \pm 0.022$	$0.894 \pm 0.012$	$0.813 \pm 0.015$	$0.887 \pm 0.005$
Mask	Resnet	$0.766 \pm 0.015$	$0.843 \pm 0.016$	$0.728 \pm 0.015$	$0.813 \pm 0.015$
Texture	Resnet	$0.788 \pm 0.021$	$0.861 \pm 0.018$	$0.753 \pm 0.036$	$0.843 \pm 0.014$
**FreeSurfer**					
Point cloud	PointNet++	$0.790 \pm 0.015$	$0.864 \pm 0.009$	$0.780 \pm 0.011$	$0.849 \pm 0.009$
Mask	Resnet	$0.787 \pm 0.014$	$0.855 \pm 0.014$	$0.754 \pm 0.029$	$0.828 \pm 0.018$
Texture	Resnet	$0.786 \pm 0.007$	$0.854 \pm 0.013$	$0.757 \pm 0.030$	$0.843 \pm 0.025$
ROI	Resnet	$0.811 \pm 0.012$	$0.882 \pm 0.021$	$0.765 \pm 0.006$	$0.857 \pm 0.015$

Top: Segmentations obtained using FSL FIRST software. Bottom: Segmentations obtained using FreeSurfer software.

**Table 2:** Time-to-dementia prediction performance on ADNI for different representations.

Representation	Network	c-index
**FSL**		
Point cloud	PointNet++	$0.572 \pm 0.024$
Mesh	SpiralNet++	$0.629 \pm 0.036$
Mask	Resnet	$0.597 \pm 0.036$
Texture	Resnet	$0.583 \pm 0.029$
**FreeSurfer**		
Point cloud	PointNet++	$0.592 \pm 0.042$
Mask	Resnet	$0.630 \pm 0.029$
Texture	Resnet	$0.610 \pm 0.031$
ROI	Resnet	$0.627 \pm 0.016$

Top: Segmentations obtained using FSL FIRST software. Bottom: Segmentations obtained using FreeSurfer software.

### Dementia diagnosis prediction

Table 1 reports the classification accuracy of the different representations for processing the images with FSL FIRST and FreeSurfer, respectively. We report the accuracy for ADNI and AIBL, where only ADNI has been used for training and the AIBL results therefore indicate the generalization to an independent test set.

Focusing first on FSL results, we observe that SpiralNet++ with meshes yields higher accuracy than point cloud, mask or texture on ADNI. The addition of texture helps the diagnosis prediction, as shown by the higher accuracy compared to mask. Point clouds had a slightly worse accuracy than masks in this experiment. The AIBL results show a similar ordering of the different representations, but meshes have the smallest decrease in accuracy by just 1.1%, while the other methods decrease between 2.8 and 3.8% in accuracy.

Point clouds and masks extracted from FreeSurfer segmentations perform much better than their counterparts from FSL. Particularly, the accuracy of point clouds improve by more than 3% on ADNI and more than 4% on AIBL. These results indicate that more Alzheimer’s specific information is contained in the FreeSurfer segmentations than in the ones from FSL. The results for texture are comparable for both processing streams. Volumetric ROI has the second best performance on ADNI, but the accuracy drops by 4.6% on AIBL. The high accuracy of the ROI is expected, since it holds information about neighboring structures (see table S3), while the rest of the models only have access to the hippocampus.

### Time-to-dementia prediction

The results for time-to-dementia prediction are summarized in Table 2. Overall, the performance of all models is relatively poor ranging between a mean c-index of 0.583 to 0.630, which is accompanied by a relatively high variance across folds. We can observe agreement with the results on dementia diagnosis in two experiments. First, the mesh-based network outperforms all competing methods by a considerable margin on the FSL data (0.032 higher mean c-index). Second, the point cloud network performs considerably worse on point clouds derived from FSL segmentations than on those derived from FreeSurfer segmentations (0.02 lower mean c-index). The results on volumetric texture are strikingly different for time-to-dementia prediction, where including texture information degrades performance compared to using a volumetric mask. When using a volumetric ROI, the gap becomes narrower, but the simpler volumetric mask representation is preferred in terms of mean performance (0.630 vs. 0.627 mean c-index), but suffers from almost double the variance across folds. Finally, the performance based on volumetric masks is comparable across FSL and FreeSurfer data, which is in contrast to the results on dementia diagnosis.

**Figure 3:**
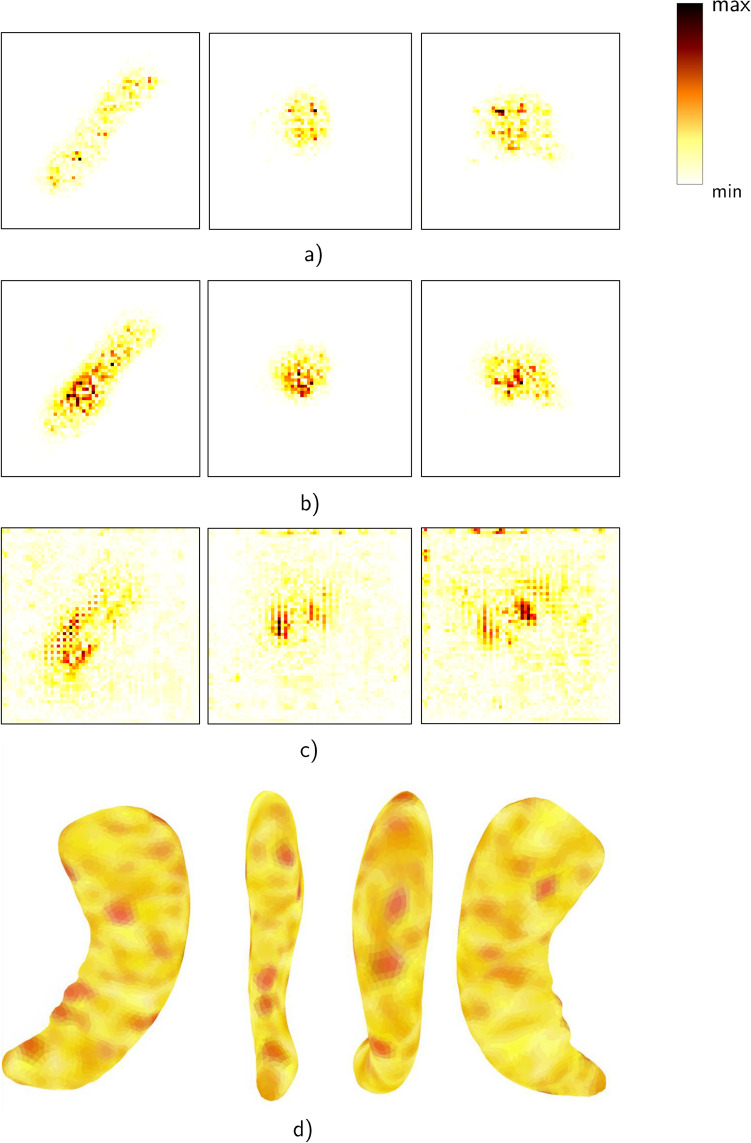
Average of integrated gradients computed across the AD population for the volume (**a**–**c**) and mesh representations (**d**). (**a**–**c**) Shown for only on slice in sagital, coronal and axial (respectively): (**a**) volumetric mask, (**b**) texture, and (**c**) ROI. (**d**) Views (from left to right): superior, medial, lateral and inferior

**Table 3:** Effect of augmentation for volumetric representations (BACC: balanced accuracy, AUC: Area under the ROC curve) on ADNI and AIBL, as well as, the concordance index (c-index) for ADNI.

Repr	Aug	ADNI BACC	ADNI AUC	AIBL BACC	AIBL AUC	c-index
**FSL**						
Mask	$\times$	$0.766 \pm 0.015$	$0.843 \pm 0.016$	$0.728 \pm 0.015$	$0.813 \pm 0.015$	$0.597 \pm 0.036$
Mask	$\checkmark$	$0.801 \pm 0.011$	$0.869 \pm 0.007$	$0.765 \pm 0.016$	$0.853 \pm 0.006$	$0.623 \pm 0.017$
Texture	$\times$	$0.788 \pm 0.021$	$0.861 \pm 0.018$	$0.753 \pm 0.036$	$0.843 \pm 0.014$	$0.583 \pm 0.029$
Texture	$\checkmark$	$0.819 \pm 0.014$	$0.888 \pm 0.007$	$0.795 \pm 0.019$	$0.870 \pm 0.010$	$0.631 \pm 0.028$
**FreeSurfe**r						
Mask	$\times$	$0.787 \pm 0.014$	$0.855 \pm 0.014$	$0.754 \pm 0.029$	$0.828 \pm 0.018$	$0.630 \pm 0.029$
Mask	$\checkmark$	$0.803 \pm 0.011$	$0.879 \pm 0.008$	$0.781 \pm 0.012$	$0.868 \pm 0.004$	$0.646 \pm 0.037$
Texture	$\times$	$0.786 \pm 0.007$	$0.854 \pm 0.013$	$0.757 \pm 0.030$	$0.843 \pm 0.025$	$0.610 \pm 0.031$
Texture	$\checkmark$	$0.808 \pm 0.011$	$0.886 \pm 0.006$	$0.783 \pm 0.014$	$0.864 \pm 0.005$	$0.657 \pm 0.037$
ROI	$\times$	$0.811 \pm 0.012$	$0.882 \pm 0.021$	$0.765 \pm 0.006$	$0.857 \pm 0.015$	$0.627 \pm 0.016$
ROI	$\checkmark$	$0.850 \pm 0.004$	$0.916 \pm 0.007$	$0.817 \pm 0.019$	$0.892 \pm 0.016$	$0.637 \pm 0.037$

### Data augmentation

Given that the amount of data is limited for Alzheimer’s Disease prediction tasks, especially when it comes to time-to-dementia prediction, we want to explore the effect of augmentation strategies during training. We randomly apply rotation (maximum of 45 degrees) and translation (maximum of 6 mm) to the volumetric representations (mask, texture and bounding box) for online augmentation. Notice that these techniques are only useful for the image representations, since PointNet++ and SpiralNet++ do not benefit from them. PointNet++ is rotation and translation invariant by design. SpiralNet++ relies on the precomputed operations on the template, hence, if these transformations are applied, the input shape would not be registered anymore.

Table 3 shows that augmentation yields an improvement on every metric for every representation. The classification accuracy for ROI with augmentation is highest, outperforming SpiralNet++. However, the accuracy on AIBL drops by 3.3%, so that is only 0.4% above SpiralNet++, while having a higher variance. The best results for prognosis prediction are obtained by texture from FreeSurfer. Also the prediction from the mask is better than ROI for this task. These results indicate that shape information is more important for prognosis than for diagnosis when augmentation is considered.

### Post-hoc explanation via relevance maps

Computing relevance maps is a helpful way of assessing the decision-making process of a classification model, since it allows us to know which areas of the hippocampus are more affected when developing the disease. We used Integrated Gradients (IG)^39^ for computing the gradient of the model’s prediction output to its input features. Given an input representation and a baseline (which is defined by the user), IG creates a set of interpolated inputs between the two. The final contribution is calculated through averaging the gradients of all the interpolations. Since our focus is on understanding the main areas of the hippocampus that get affected by Alzheimer’s disease, we compute the IG for all the patients diagnosed with AD and average the individual saliency maps to obtain a final one describing the population. Note that this type of analysis cannot be applied to point clouds given its lack of order in the input points. As baseline for the mesh, we randomly selected a CN patient, since we are only interested in the contributions of the AD population. For the volumetric representations (volumetric mask, texture and bounding box), we define a black 64 × 64 × 64 cube as baseline.

Figure 3a–c shows the relevance maps for volumetric representations: mask, texture, and ROI. The texture highlights more regions in the interior of the hippocampus than the mask. From the ROI, we observe that also regions outside of the hippocampus are relevant, but the focus is on the hippocampus. Overall, it is difficult to clearly identify relevant regions from these maps, as there is no one-to-one correspondence between images so that averaging yields blurring of the relevant regions. In contrast, saliency maps on meshes, see Fig. 3d, do allow for a more detailed analysis of relevant regions, because correspondences between all meshes exist. Thus, meshes not only show the geometry of the hippocampus but also offers a more granular representation of the important regions.

## Discussion

In this work, we have compared five representations of the hippocampus for the prediction of Alzheimer’s disease. Based on our results, the choice of the most suitable representation will come down to the application’s requirements. A common criterion is the *performance* yielded by the combination of the representation and its associated network for a given task. We evaluated the capabilities of each representation–network pair for predicting Alzheimer’s disease diagnosis and the time to dementia. On both tasks, meshes with SpiralNet++ provide the highest performance. However, volumetric representations follow a grid-like structure and therefore can directly benefit from advances in CNNs, such as data augmentation (section “Data augmentation”). When adding on-the-fly augmentation, the volumetric ROI and texture yield the best results for diagnosis and time-to-dementia prediction tasks (Table 3), respectively. In addition, the former requires the *least pre-processing*, since it only extracts a 3D bounding box around the hippocampus without requiring an accurate segmentation.

The volumetric ROIs do not only include the hippocampus, but also neighboring structures. Table S3 shows that the hippocampus has the highest relative importance, but also the other structures contribute to the prediction. While this leads to high performance, the results are not directly comparable to the other representations and it is less suited if the focus should be on studying hippocampus’ atrophy due to dementia. For that application, shape representations, i.e., point clouds, meshes, and volumetric mask, are more suitable since the network predictions are exclusively driven by changes in their structure without taking into account the texture information. In particular, mesh-based SpiralNet++ is the best representation-method combination for this type of application—provided a template is available, as it is the case for segmentations using FSL.

We also explored the ability for the models to *generalize* to unseen data. Studies can differ in the image acquisition protocol and also in the population composition, which can complicate the transfer of models. Representations that only depend on intensity values like volumetric ROIs are more affected by acquisition variations, than those that rely on shape representations that present a higher level of abstraction. Besides high performance in the prediction tasks, meshes also provide the highest granularity when running *interpretability* algorithms, producing heat maps that allow us to perform subfield analysis. The areas that are highlighted in Fig. 3d) are the medial part of the body in the subiculum and parasubiculum areas, the lateral part of the body in the CA1 area and the inferior part of the hippocampus head in the subiculum area. Those areas correspond to previous findings in neuroscience research^40^. These findings provide great clinical value, since it can help building trust on the model decisions. Yet, a reference template is not always available, e.g., for FreeSurfer segmentations. In those cases, point clouds also provide a light weight representation and perform better than volumetric masks. Finally, although ADNI provides the largest neuroimaging resource for AD, the sample size is still orders of magnitude smaller than large-scale computer vision datasets. Hence, the amount of training data is limited, so that networks with a *low number of learnable parameters* are very relevant. SpiralNet++ approaches this issue by pre-defining the convolution sequences and pooling operations on the template, which besides decreasing run time, also can help reducing the number of parameters.

In our study, we have paid particular attention on a rigorous evaluation of the different representations. We carefully split the data in training, validation and test set, to avoid data leakage and confounding bias (see section “Data stratification”). It was recently noted that many studies for AD prediction with deep learning are subject to one or more sources of data leakage^11^, which can inflate the reported results. We resolved bias by splitting data so that age, sex, and education distributions match across splits (see section “Data stratification”). Next to carefully partitioning the data, we have also reported results on an independent test set to ensure reproducibility.

Our results indicate that *Time-to-dementia prediction* is more challenging than AD Diagnosis. In the former, images are taken at baseline from which we want to predict the time of dementia onset, which can be a decade in the future (see Fig. 2). The median time of MCI to dementia progression is 5 years in ADNI. However, neuroanatomical changes, as captured by structural MRI scans, manifest in the late stages of AD, which makes predictions for patients at the early stage of AD extremely challenging. In addition, these neuroanatomical changes are mostly related to structural atrophy and texture carries little additional information. Finally, we would like to point out that the path to dementia is highly heterogeneous and neuroanatomical changes are only one piece of the puzzle. Therefore, a clinically useful model for time-to-dementia prediction has to incorporate multi-modal data^29^. Our study provides valuable information about the best representation to augment with additional clinical information.

We note that the selection of a representation also implies the selection of a particular set of network architectures (e.g., meshes cannot be processed with regular CNNs). While this can limit the one-to-one comparison between representations, we believe that by choosing the state-of-the-art architecture for each representation, we obtained a fair comparison of the predictive performance of each hippocampus representation.

In conclusion, our findings support that mesh-based representations of the hippocampus are the preferred choice in terms of ease of interpretability. However, these are outperform by ROIs when augmentation is considered. Considering that AD is a multifactorial and heterogenous diseases, future research should focus on multi-modal DL approaches. Our work highlights that how data is represented can have far-reaching consequences on performance and interpretability. This will likely be even more important when combining multi-modal data and should be at the center of future multi-modal approaches. In addition, we believe the obtained results could be extrapolated to other applications, such as the analysis of other organs or even objects. The objectiveness of the experimental set up can help a better understanding of the benefits and drawbacks of each representation when evaluated under the exact same conditions.

## Methods

### Networks

For each representation, we selected the current state-of-the-art architecture for Alzheimer’s disease diagnosis according to previously published results.

#### Point cloud network

The main idea of PointNet++ is to capture features at increasingly larger scales along a multi-resolution hierarchy. For every hierarchy level *h*, three main operations are defined: sampling, grouping, and feature extraction.

The input of each hierarchical level will be the coordinates of the points, i.e., centroids of the previous hierarchical level, ${\mathbf {P}}^{h-1}= \{{\mathbf {p}}_1^{h-1},\ldots,{\mathbf {p}}_{N^{h-1}}^{h-1}\}$

and point cloud descriptors ${\mathbf {F}}^{h-1}$ composed of the local point features extracted from the previous hierarchical level ${\mathbf {F}}^{h-1}= [{\mathbf {f}}_1^{h-1},\ldots,{\mathbf {f}}_{N^{h-1}}^{h-1}]$ with ${\mathbf {f}}_i^{h-1} = [c_1^{h-1},\ldots,c_{C^{h-1}}^{h-1}]$ being the point feature associated to the ${\mathbf {p}}_i^{h-1}$ centroid’s neighborhood and $c_j$ its *j*-th channel.

##### Sampling

In this step, $N^{h}$ points from the input ${\mathbf {P}}^{h-1}$ (with $N^{h} \le N^{h-1}$) are sampled and used as centroids of the pooling regions for the next hierarchical level. The sampling method must be invariant to the order of the points, hence^32^, proposes farthest point sampling (FPS).

##### Grouping

Once the $N^{h}$ centroids have been sampled, the next step is to define their respective neighborhoods. As shown in^32^, selecting a *K* number neighbors is not as effective as query ball sampling (given a radius), especially if the points are not equally distributed along the point cloud. The radius of the query ball increases for each hierarchical level, emulating the receptive field of filters in a CNN. Note that the number of samples for each centroid can vary as the feature network maps sets with different number of points into a fixed length feature vector.

##### Feature extraction

Finally, given $N^{h}$ point coordinates and their associated point features extracted in the previous level, the coordinates of the points in each local region are first expressed relative to the region’s centroid and then concatenated to the point features, ${\mathbf {F}}^{h-1}$, extracted in the previous hierarchical level. The concatenated vector is passed through a PointNet^41^, shared along $N^h$ sub-sets of point cloud descriptors in the hierarchical level, to compute the new local point features ${\mathbf {f}}_i^{h}$ that form the new point cloud descriptors ${\mathbf {F}}^{h}$. Notice that in $h=1$, $C^0=3$ since the point features will be the zero-centered coordinates of points inside the query balls.

#### Mesh network

For learning on meshes, we use the recently introduced SpiralNet++^33^, which has achieved state-of-the-art performance in several computer vision tasks, as well as, for Alzheimer’s detection^25^. SpiralNet++ proposed a novel *message passing* approach to deal with irregular representations like meshes. The encoder blocks of SpiralNet++ are formed by two main operations: spiral convolution and mesh pooling.

##### Spiral convolution

Due to the nature of triangular meshes, a spiral serialization of neighboring nodes is possible. Given a vertex in *V*^33^, defines its spiral sequence by choosing an arbitrary starting direction in counter-clockwise manner. Figure 1 illustrates a spiral sequence. In comparison to SpiralNet^42^, SpiralNet++ defines these sequences only once for a template shape and then applies them to the aligned samples in the dataset, highly increasing the efficiency of the method.

The convolution operation in layer *k* for features ${\mathbf {x}}_i$ associated to the *i*-th vertex is therefore defined as:

1 $$\begin{aligned} {\mathbf {x}}_i^{(k)} = \gamma ^{(k)}\left( \underset{j \in S(i,l)}{\parallel } {\mathbf {x}}_j^{(k-1)} \right) , \end{aligned}$$

where $\gamma$ denotes *multi-layer-perceptron* (MLP) and $\parallel$ is the concatenation operation. *S*(*i*, *l*) is an ordered set consisting of *l* vertices inside the spiral.

##### Mesh pooling

The down-sampling operation or *pooling* is obtained by iteratively contracting vertex pairs that would minimize the quadric error^43^. In Fig. 1, we illustrate this process. For efficiency, the coordinates of the vertices that must be pooled in each level are computed for the template and then applied to the samples in the dataset.

More details about the method and its implementation can be found in^33^.

#### Convolutional neural network

For volumetric representations, one can draw upon the pool of convolution neural networks. In Table S4, we compare a regular ConvNet, like the one proposed in^11^, to a 3D version of the ResNet^34^ (depicted in Fig. 1). The latter outperformed the former and therefore was selected for all the experiments on the volumetric representations. It comprises ten convolutional layers with kernel size $3^3$ followed by batch normalization^44^ and rectified linear unit (ReLU) activation. We half the spatial resolution of the feature map in the second convolutional layer by using a stride of 2. It is followed by one residual block without downsampling, and two more residual blocks with downsampling. Finally, we perform global average pooling across the spatial resolution of the feature maps and use a linear layer to output a log-probability.

### Study population

#### Dementia diagnosis

For dementia diagnosis, we restricted data to the baseline visit and included all patients that have been diagnosed as either healthy control or as demented, for which age and gender have been recorded, and for which a MRI scan was available and produced a valid segmentation with FreeSurfer^30^ and FSL FIRST^31^. This resulted in a total of 1505 patients for ADNI, and 552 patients for AIBL. The overall data set characteristics are summarized in Table S1.

#### Time-to-dementia progression

For analyzing time-to-dementia progression, we included all patients that were diagnosed with mild cognitive impairment (MCI) at baseline, had at least one follow-up visit, and remained MCI or progressed to dementia during the entire follow-up period, i.e., patients with bidirectional change in diagnosis were excluded. As for diagnosis, only patients for which an MRI scan was available and produced a valid segmentation with FreeSurfer and FSL FIRST were included. The time to progression was defined as the time difference between the first visit with MCI diagnosis and the first visit with dementia diagnosis. If patients were not diagnosed as demented during their entire follow-up period, we considered their time-to-progression as right censored and used the time of the last follow-up visit as time of censoring. In total we included 795 patients. Table S2 summarizes the patient characteristics.

#### Data stratification

Fair evaluation of methods is a non-trivial task in neuroimaging, as several sources of data leakage can lead to biased performance estimates. We implemented a 5-fold cross-validation scheme that avoids four common sources of bias highlighted by Wen et al.^11^: (i) the use of multiple scans per subject, (ii) applying data augmentation before data splitting, (iii) overfitting on the test set, and (iv) differences in the distribution of age, sex, and education across folds (more details .

To address issue (i), we only considered data from baseline visits in our experiments such that only a single scan per patient is included. Issue (ii) is prevented by applying data augmentation exclusively to the training portion of the data, and issue (iii) by selecting the model with highest performance on the validation set and reporting its final performance on the test set, where each fold is used once as test data and the four remaining folds are combined such that 80% of it comprise the training set and 20% the validation set.

Finally, we resolve bias due to issue (iv), by splitting data in a manner that ensures age, sex, and education distributions match across splits. To this this end, we assessed the balance of a split by computing the propensity score, i.e. the probability of a sample belonging to the training data, based on a logistic regression model comprising age, sex, and education. Next, we compared the percentiles of the propensity score distribution in the training and test data and used the maximum deviation across all percentiles as a measure of imbalance^45^. For all experiments, we account for differences in age and sex, because they are known confounders in neuroimaging studies^46^. For time-to-dementia prediction, we additionally account for differences in education, which is a proxy for cognitive reserve that is affecting the rate of progression^47^. For each of the 5 splits, this process has been repeated for 1000 randomly selected partitions and the partition with the with minimum imbalance was ultimately the selected split. More details of the data distribution can be found in Fig. 2.

### Data processing

### Image segmentation

We processed T1-weighted brain MRI scans from The Alzheimer’s Disease Neuroimaging Initiative (ADNI^37^) and The Australian Imaging, Biomarker & Lifestyle Flagship Study of Ageing (AIBL^38^) using the following procedure. First, we conformed scans to $1\,\text {mm}^3$ isotropic voxel size and a resolution of $256 \times 256 \times 256$. (Using FreeSurfer’s mri_convert--conform^30^) Next, we applied bias field correction using the N4ITK algorithm^48^, and registered the resulting image to the MNI space with the ICBM 2009c non-linear symmetric template^49,50^ using the SyN algorithm for affine registration as implemented in ANTs^51^. Our processing pipeline is similar to the minimal processing pipeline in^11^.

To obtain shape representations of the left hippocampus, we segmented pre-processed scans using FreeSurfer^30^ and FSL FIRST^31^. FreeSurfer is an atlas-based segmentation algorithm, whereas FSL FIRST employs an active appearance model to incorporate intensity information and a shape-based prior about likely variations in anatomical shape. We manually inspected all segmentations and excluded instances where either FreeSurfer or FSL FIRST failed.

#### Hippocampus representations

After segmentation, we extracted five hippocampus representations: meshes, point clouds, volumetric masks, volumentric textures, and volumentric ROIs. The details differ depending on the segmentation algorithm and are detailed below.

FreeSurfer produces a voxel-wise segmentation map from which we reconstructed a 3D surface using the marching cubes algorithm^52^, for which we adjusted the vertex coordinates using Laplacian smoothing. The resulting surfaces can have an arbitrary number of vertices, and there are no cross-subject correspondences.

FSL FIRST directly produces a 3D triangular mesh of 732 vertices for the left Hippocampus such that cross-subject correspondences of vertices are maintained. We refined the mesh by subdividing the surface into 2922 vertices and 5840 faces using the Butterfly Scheme^53^.

To obtain volumetric representations, we defined a bounding box with $64^3$ voxels in MNI space around the left hippocampus based on FreeSurfer and FSL FIRST segmentations. The same bounding box is used for all three volumetric representations. To create volumetric masks, we either used FreeSurfer’s segmentation map or FSL FIRST’s volumetric output after boundary correction. For volumetric textures, only the image intensities inside hippocampus region are used and everything else is set to zero. For volumetric ROI, the MRI scan is cropped to the bounding box, so there is no difference for this representation between FreeSurfer and FSL.

#### Data transformation

We trained our mesh network based on meshes produced by FSL FIRST (see section “Image segmentation”), where we adjusted vertex coordinates of each mesh by centering the mesh at the origin and scale it by the maximum vertexwise Euclidean distance to the origin, such that all meshes fit into the unit sphere. Point cloud networks were trained on point clouds derived from meshes produced by FreeSurfer and FSL FIRST, respectively (see section “Image segmentation”). The point clouds have the same vertex coordinates as the meshes, but do not account for connectivity. Since point clouds from FreeSurfer and FSL FIRST have different number of points, we subsampled each point cloud to 1,024 vertices (uniformly random). Analogous to meshes, we centered each point cloud at the origin and scaled it to fit into the unit sphere. The CNN was trained with ROIs of size $64 \times 64 \times 64$ around the left hippocampus as described in section “Image segmentation”. Voxel intensities were normalized to the range [0, 1] based on the min and max values of each individual image.

### Training strategy

All the models were trained to minimize the Cross-entropy loss for diagnosis prediction, and the Cox loss^54^ for time-to-dementia prediction. We used Adam^55^ as the optimization algorithm with a learning rate of 0.001. We did not observe a significant difference when trying to optimize the learning rate for each shape representation, so we decided to fix it for a fairer comparison. The batch size was set to 20, and the training duration to 200 epochs. In order to avoid over-fitting, the model with highest performance on the validation set is evaluated on the test set for reporting the final results (Validation curves can be found in Fig. S1).

## Supplementary Information

Supplementary Information.

## Data Availability

Alzheimer’s Disease Neuroimaging Initiative (ADNI) and Australian Imaging, Biomarker & Lifestyle Flagship Study of Ageing (AIBL) used in this study were available at the databases (http://adni.loni.usc.edu/) and (http://aibl.csiro.au/), respectively, upon registration and compliance with the data usage agreement.
