# Supplementary material for: Hippocampal representations for deep learning on Alzheimer’s disease
Source: Sci Rep. 2022 May 21;12:8619. doi: 10.1038/s41598-022-12533-6 (PMC9124220; doi:10.1038/s41598-022-12533-6)
Supplement: Supplementary file 1 — Supplementary Information. [file 41598_2022_12533_MOESM1_ESM.pdf]

Supplementary Material: Hippocampal Representations for Deep Learning on Alzheimer's Disease

Ignacio Sarasua, Sebastian Pölsterl, and Christian Wachinger, for the Alzheimer's Disease Neuroimaging, and the Australian Imaging Biomarkers and Lifestyle flagship study of ageing

Artificial Intelligence in Medical Imaging (AI-Med),
Department of Child and Adolescent Psychiatry,
Ludwig-Maximilians-Universität,
Waltherstr. 23, 80337 Munich, Germany

March 24, 2022

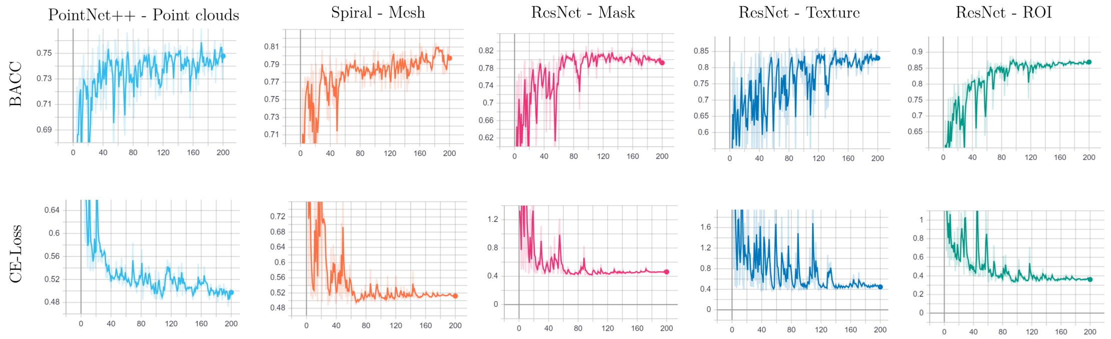

Figure S1: Cross-Entropy (CE) Loss and Balanced Accuracy (BACC) validation curves for each representation-network pair

Table S1: Summary of data used for dementia diagnosis from ADNI (top) and AIBL (bottom).

	Dementia (%)	Male (%)	Age (years)
Overall	44.6	50.3	73.4 ± 7.0
Fold 1			
train	44.6	50.1	73.4 ± 7.0
valid	44.6	50.8	73.2 ± 6.9
test	44.7	50.7	73.5 ± 7.2
Fold 2			
train	44.6	49.8	73.4 ± 7.1
valid	44.6	51.7	73.2 ± 6.9
test	44.7	50.7	73.4 ± 6.8
Fold 3			
train	44.6	49.8	73.3 ± 7.0
valid	44.6	51.2	73.5 ± 7.0
test	44.7	51.0	73.4 ± 7.1
Fold 4			
train	44.7	50.5	73.4 ± 7.1
valid	44.6	50.4	73.4 ± 6.8
test	44.3	49.7	73.3 ± 7.1
Fold 5			
train	44.6	50.5	73.4 ± 7.1
valid	44.6	50.4	73.4 ± 6.9
test	44.6	49.5	73.2 ± 7.0
AIBL	13.8	42.0	72.6 ± 6.4

Table S2: Summary of ADNI data used for time-to-dementia prediction.

	Follow-up (years)	Progressed (%)	Male (%)	Age (years)	Education (years)
Overall	2.8 ± 2.2	36.9	60.0	73.7 ± 7.2	15.9 ± 2.9
Fold 1					
train	2.7 ± 2.1	36.9	59.8	73.6 ± 7.2	15.9 ± 2.9
valid	2.9 ± 2.2	37.0	60.6	73.8 ± 7.1	15.9 ± 2.9
test	2.8 ± 2.3	36.7	60.1	73.8 ± 7.5	15.9 ± 2.8
Fold 2					
train	2.8 ± 2.2	36.9	60.0	73.8 ± 7.2	15.9 ± 2.9
valid	2.8 ± 2.3	37.0	59.1	73.5 ± 7.3	15.9 ± 2.9
test	2.7 ± 2.1	36.7	60.8	73.5 ± 7.2	15.9 ± 2.9
Fold 3					
train	2.8 ± 2.1	36.9	60.0	73.7 ± 7.3	15.9 ± 3.0
valid	2.8 ± 2.4	37.0	59.8	73.8 ± 7.3	15.9 ± 2.7
test	2.7 ± 2.2	36.7	60.1	73.4 ± 7.1	15.9 ± 2.8
Fold 4					
train	2.8 ± 2.2	36.9	60.6	73.6 ± 7.3	15.9 ± 2.8
valid	2.8 ± 2.2	37.0	59.1	73.6 ± 7.2	15.9 ± 2.8
test	2.7 ± 2.2	36.7	58.9	74.0 ± 7.3	15.9 ± 3.1
Fold 5					
train	2.7 ± 2.2	36.8	59.7	73.6 ± 7.3	15.9 ± 2.9
valid	2.8 ± 2.2	36.5	61.1	73.8 ± 7.2	15.9 ± 2.9
test	2.9 ± 2.0	37.4	60.1	73.7 ± 7.2	15.9 ± 2.8

Table S3: Analysis of the importance of the different anatomical structures that are present in the ROI around the left hippocampus. The table lists the size of the structures, their importance for dementia prediction as measured with integrated gradients (IG), and the importance normalized by size (used for sorting).

Structure	Average Size	Average IG	Normalized avg IG
Left-Hippocampus	1.799	3.985	2.216
Left-Inf-Lat-Vent	0.599	0.769	1.283
Left-Amygdala	0.656	0.776	1.183
Left-Pallidum	0.742	0.835	1.126
Left-VentralDC	1.815	1.923	1.059
Left-Putamen	1.786	1.754	0.982
Left-Cerebral-Cortex	17.055	15.703	0.921
Left-Cerebral-White-Matter	19.911	17.725	0.890
Optic-Chiasm	0.103	0.089	0.858
Left-Cerebellum-Cortex	5.156	4.020	0.780
Left-Thalamus-Proper*	3.486	2.357	0.676
Left-Cerebellum-White-Matter	3.673	2.425	0.660
WM-hypointensities	0.191	0.123	0.644
Brain-Stem	6.070	3.796	0.625
Unknown	27.442	15.863	0.578
Left-choroid-plexus	0.798	0.448	0.562
CC-Posterior	0.233	0.098	0.421
CSF	0.671	0.278	0.414
Left-Caudate	0.351	0.143	0.408
Right-VentralDC	0.290	0.117	0.402
3rd-Ventricle	0.907	0.347	0.383
Right-Cerebellum-Cortex	0.698	0.247	0.354
Right-Cerebellum-White-Matter	0.258	0.079	0.306
Right-Cerebral-White-Matter	0.308	0.094	0.306
Left-Lateral-Ventricle	3.335	0.906	0.272
4th-Ventricle	0.701	0.179	0.256
Right-choroid-plexus	0.173	0.041	0.237
Right-Thalamus-Proper*	0.229	0.051	0.221
Right-Cerebral-Cortex	0.187	0.039	0.210
Right-Lateral-Ventricle	0.264	0.025	0.096

Table S4: Comparison of network architectures based on FreeSurfer segmentations.

Network	Representation	BACC	AUC
PointNet	Point Cloud	0.770 ± 0.018	0.850 ± 0.015
PointNet++	Point Cloud	0.790 ± 0.015	0.864 ± 0.007
DGCNN	Point Cloud	0.781 ± 0.004	0.845 ± 0.005
ConvNet	Mask	0.759 ± 0.011	0.822 ± 0.014
ResNet	Mask	0.787 ± 0.012	0.855 ± 0.011
ConvNet	Texture	0.762 ± 0.011	0.813 ± 0.011
ResNet	Texture	0.786 ± 0.007	0.854 ± 0.011
ConvNet	ROI	0.787 ± 0.009	0.855 ± 0.009
ResNet	ROI	0.811 ± 0.013	0.882 ± 0.014

Table S5: Comparison of network architectures based on FSL segmentations.

Network	Representation	BACC	AUC
PointNet	Point Cloud	0.747 ± 0.013	0.817 ± 0.017
PointNet++	Point Cloud	0.755 ± 0.012	0.820 ± 0.012
DGCNN	Point Cloud	0.738 ± 0.017	0.808 ± 0.014
ConvNet	Mask	0.745 ± 0.021	0.808 ± 0.019
ResNet	Mask	0.766 ± 0.012	0.843 ± 0.011
ConvNet	Texture	0.758 ± 0.022	0.847 ± 0.012
ResNet	Texture	0.788 ± 0.016	0.861 ± 0.012

Table S6: Number of parameters of the different networks

Network	Number of Paremeters
PointNet++	$1,465,665$
SpiralNet++	$96,529$
Resnet	$897,313$
